# Efficacy of Iodine-125 Seed Implantation in Locoregionally Recurrent and Unresectable Breast Cancer: a Retrospective Study

**DOI:** 10.1007/s12253-017-0361-9

**Published:** 2017-11-07

**Authors:** Ying-hua Yu, Chang-yuan Wei, Qing-hong Qin, Qin-guo Mo, Zhen Huang, Bin Lian

**Affiliations:** Departmant of Breast Surgery of Affiliated Tumor Hospital of Guangxi Medical University, NO. 71, He Di Lu, Nanning, Guangxi 530021 People’s Republic of China

**Keywords:** Iodine-125 seed implantation, Brachytherapy, Locoregionally, Unresectable, Breast cancer

## Abstract

The management of locoregionally recurrent and unresectable breast cancer is a therapeutic challenge. This retrospective study aimed to assess the efficacy of ^125^I seed implantation brachytherapy as a palliative management in locoregionally recurrent breast cancer. We analyzed 36 locoregionally recurrent and unresectable breast cancers in our hospital between 2012 and 2016. All patients were treated with CT-guided ^125^I seed permanent implantation. The dose distribution of ^125^I seeds was calculated using a computerized treatment planning system. Complete response, partial response, stable disease, and local tumor control rates were calculated. Long-term efficacy was assessed based on survival rates ranging from 1 to 4 years. The follow-up period ranged from 6 to 53 months. The median local control was 28 months (95% CI: 16.2–39.8 months). The percentage of patients who showed 6-month, 1-year, 2-year, and 3-year local control was 97.2%, 77.8%, 52.8%, and 33.3%, respectively. Median survival time for all patients was 48 months (95% CI: 40.9–55.1 months); 1-year, 2-year, 3-year, and 4-year survival rates were 97.2%, 80.6%, 63.9%, and 46.5%, respectively. Pain relief response rate was 88.9%. No serious complications were detected during the follow-up period. The results of this study demonstrate that ^125^I seed implantation could be considered a feasible and promising minimally invasive therapy for locoregionally recurrent and unresectable breast carcinoma.

## Introduction

The number of patients experiencing locoregionally recurrent breast cancer (LRBC) has been increasing steadily over the past decade. After mastectomy, 2% to 31% of patients suffer from a second local recurrence [1]. Even after adjuvant radiation therapy (RT), the rate of locoregional recurrences remains around 5% to 15% [2, 3]. Almost 80% of these recurrences appear usually within the first 5 years from the primary treatment [4], and approximately 10% of them are represented by inoperable breast cancer. Therefore, a promising palliative therapy should be considered for these patients.

The management of LRBC is a therapeutic challenge. The most common therapies involve surgery, external beam RT (EBRT), chemotherapy, or hormonal interventions [5]. However, these treatments still have their limitations. Some of the locally recurrent breast tumors cannot be completely removed. Chemotherapy failed to achieve therapeutic benefits, and some recurrent breast cancer patients are not responding to endocrine therapy, as shown by a systematic review of randomized trials [6]. Excessive external beam radiotherapy could increase the probability of both acute and late toxicities, because of the risk of exceeding the radiation tolerance limits. Therefore, another palliative therapy should be considered.

Recently, image-guided iodine-125 (^125^I) seed brachytherapy has received more and more attention, several studies reporting that ^125^I seed brachytherapy provided promising results for the local control of solid tumors such as head and neck cancers, prostatic malignancies, and many other malignant tumors [7–11]. In these studies, ^125^I seed implantation was capable of delivering a sufficient dose of radiation to the tumor mass, causing less damage to adjacent normal tissues.

Although ^125^I radioactive seeds were studied and applied in prostate cancer and other tumor types, they have been relatively neglected in LRBC treatment, being used at present for the localization of non-palpable breast tumors, or to mark the tumor bed for neo-adjuvant chemotherapy or breast-conserving surgery [12, 13]. Up to now, no reports are available investigating ^125^I seed implant brachytherapy as a multi-modality therapy of LRBC.

Hence, we performed this retrospective study to investigate the efficacy of ^125^I seed implantation in LRBC and to determine whether this therapy could be beneficial as a palliative method in this recurrent cancer.

## Materials and Methods

### Patients

Thirty-six patients, 36–86 years old (median age 46 years), with locally advanced breast cancer were enrolled in this retrospective study conducted from January 2012 to June 2016. During this period, the patients underwent computed tomography (CT)-guided ^125^I seed implantations at the Breast Center of Affiliated Cancer Hospital of Guangxi Medical University, Guangxi, China. The inclusion criteria were the following: patients enrolled in the study had a history of surgery, adjuvant chemotherapy, or hormonal interventions, with or without EBRT; unresectable and locally recurrent breast lesions were confirmed by histopathological or cytopathological examination; ultrasound or CT scan indicated a solid mass or nodule in the local area. These patients were not suitable for salvage surgery alone after being reviewed by surgeons. Among these patients, 14 had chest wall metastasis, 10 had axillary metastasis (which caused edema of the upper extremity), 8 had supraclavicular metastasis, and 4 had metastasis in other sites. The characteristics of these patients are listed in Table 1.Written informed consent was obtained from all patients, and the study was approved by the institutional ethics committee of the Affiliated Tumor Hospital of Guangxi Medical University.

**Table 1:** Patient charateristics (*n* = 36)

Characteristic	Value	Percentage(%)
Median age (range)	46(36–86)	
Primary tumor stage(n)		
stage I	2	5.5
stage II	5	13.9
stage III	19	52.8
stage IV	8	22.2
Unclear	2	5.6
Tumor site(n)		
chest wall	14	38.9
axillary	10	27.8
supraclavicular	8	22.2
other site	4	11.1
KPS(n)		
50	11	30.5
60	9	25
70	11	30.6
≥ 80	5	13.9
Previous surgery	36	100
Previous chemotherapy	34	94.4
Previous radiotherapy	34	94.4
One	3	8.3
Two	26	72.2
Three	5	13.9
Four	2	5.6

KPS = Karnofsky Performance Status score

### Treatment Process

Three days prior to ^125^I seed implantation, all patients underwent a CT scan. According to the CT image, the total volume of each tumor was calculated using a treatment planning system (TPS) [14]. The dose of ^125^I seed implantation was prescribed as the minimal peripheral dose (MPD), which encompassed the planning treatment volume (PTV). PTV included the entire gross tumor volume (GTV) and 0.5–1.0 cm margins that were outlined by the radiation oncologist on each CT transverse image.

The expected number of implanted seeds was calculated according to the modified level formula [15]. The length of ^125^I seeds (Model 6711; Beijing Atom and High Technique Industries Inc., Beijing) was 4.5 mm, and the diameter was less than 1 mm. The properties of ^125^I seeds were the following: low X-ray energy level of 27.4–31.4 KeV, a half-life (t 1/2) of 59.6 days, and a half-value of tissue penetration of approximately 1.7 cm. These properties gave a sharp drop-off dose and allowed a safe handling. Specific equipment, such as 18-G implantation needles and turntable implantation gun (XinKe Pharma-ceutical Ltd., Shanghai, China), was used. The implant parameters are listed in Table 2.

**Table 2:** Implant parameters

Characteristic	Median	Range
Volume implanted(cm3)	39	3.6–143.2
MPD (Gy)	110	90–140
Activity/seed (mCi)	0.6	0.4–0.7
Numbers of seeds	36	8–101
Total activity (mCi)	22.9	2.6–86.4
Dose rate (Gy/h)	0.07	0.05–0.09

The therapeutic intervention was performed in the CT room. All patients underwent local anesthesia before ^125^I seed implantation. The procedure time was approximately 30–160 min. The operation procedure was as follows: under CT supervision, 18-gauge interstitial needles were inserted through the skin, into the tumor lesion, within the PTV. Most of the needles were placed in a parallel array 1.0–1.5 cm apart and extended at least 0.5–1 cm beyond the GTV. Next, the ^125^I seeds were implanted using a Mick applicator. Two to six seeds per needle were loaded and released every 0.5–1 cm apart upon needle withdrawal. During the procedure, neural structures and large vascular structures were carefully avoided. The median number of ^125^I seeds used in our study was 36 (range 8–101). Median procedure time was 42 min (range 26–93 min). Puncture site was bandaged and compressed to achieve hemostasis after the procedure. Patients remained under observation at the hospital for 1 or 2 full days.

### Post ^125^I Seed Implantation

After seed implantation, 28 of the 36 patients received 4–6 cycles of chemotherapy, and eight patients received endocrine therapy. Chemotherapy and endocrine therapy were performed according to the latest guidelines of the National Comprehensive Cancer Network (NCCN).

### Clinical Effect Evaluation

All 36 patients started the follow-up phase immediately after ^125^I brachytherapy. The median follow-up was 45.9 months (range 14.9–53 months). Clinical examination, blood sampling, ultrasound, and CT examination at 1 month, 2 months, and every 3 months post-intervention in the first year were performed. Next, the above medical check-up was performed every 6 months. One case was lost during the follow-up period, after 33.6 months.

Tumor response was assessed according to World Health Organization (WHO) criteria [16]. Complete response (CR) was defined as the complete disappearance of the lesion lasting for more than 4 weeks. Partial response (PR) was defined as a decreased lesion size by more than 50% that remains stable for 4 weeks. Stable disease (SD) was defined as a tumor size change of less than 25% increase or less than 50% decrease. The sum of CR and PR was defined as response rate. Local control after ^125^I brachytherapy was defined as lack of tumor progression either in or adjacent to the implanted site. For the calculation of overall survival, deaths due to any reason were scored as events. Pain intensity was measured and graded according to the Numerical Rating Scale (NRS) of the Adult Cancer Pain Clinical Practice Guidelines [17]: 0 indicated no pain, 1–3 indicated mild pain, 4–6 indicated moderate pain, and 7–10 indicated severe pain.

### Statistical Analysis

Local control rates and overall survival (OS) were the primary endpoints of this study. Data were calculated using the Kaplan-Meier method in SPSS 13.0 software (SPSS, Chicago, USA).

## Results

### Pain Relief and Response to Treatment

Before ^125^I seed implantation, from the 36 patients, nine experienced severe pain (NRS 7–10), 19 patients had moderate pain (NRS 4–6), and eight had mild pain (NRS 1–3). Most patients reported pain relief 2–8 days after treatment. Four patients showed no change after implantation: two patients still felt moderate pain, one patient felt mild pain and one patient felt severe pain (Table 3). Pain relief response rate was 88.9%.

**Table 3:** change of pain score

	No pain (%)	Mild pain (%)	Moderate pain (%)	Severe pain (%)
Pre-treament	0(0/36)	22.2(8/36)	56.8(19/36)	25(9/36)
Post-teament	33.3(12/36)	47.2(17/36)	16.7(6/36)	2.8(1/36)

Data are presented as %(cases)

Tumor response was evaluated after repeated clinical examination, ultrasound, and CT examination. The results revealed that eight patients achieved complete response (CR), twenty-two patients achieved partial response (PR), five patients showed stable disease (SD), and progressive disease (PD) was present in one case. The overall response rate (CR + PR) in this period was 83.3%.

### Local Control and Survival

**Fig. 1:**
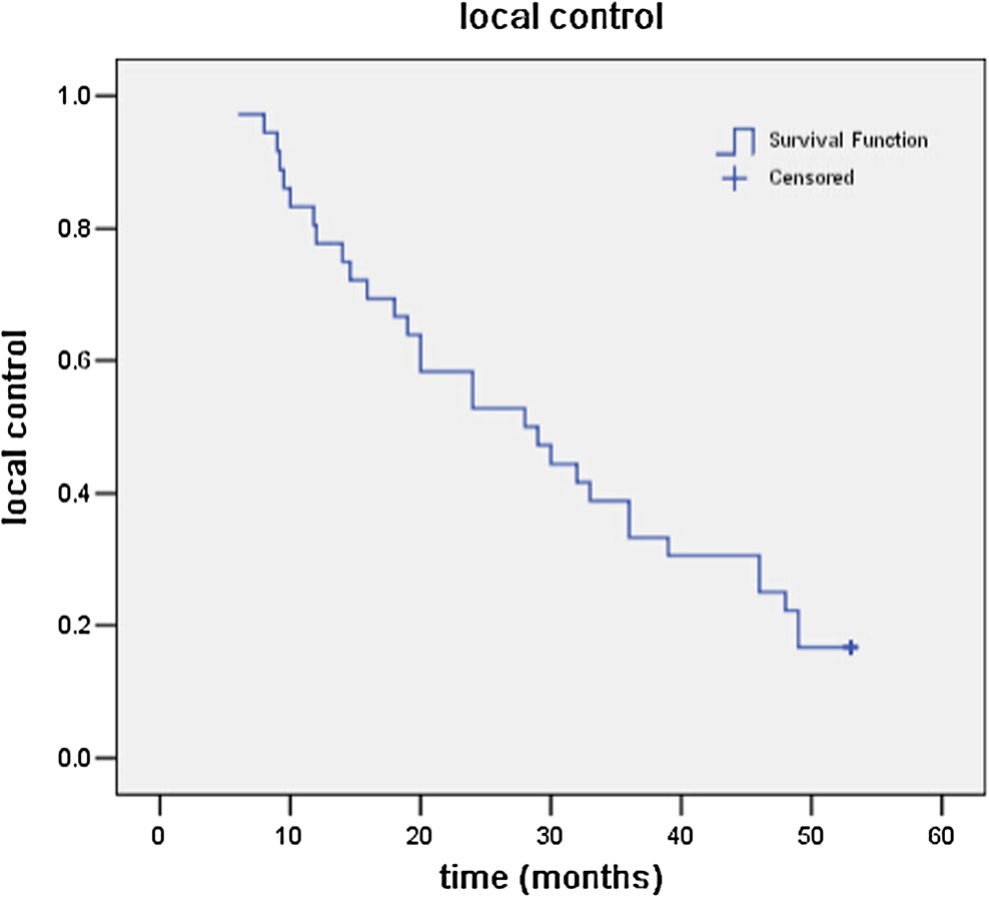
Local control for all patients after iodine-125 seed implantation therapy

The follow-up period ranged from 6 to 53 months. A locoregional failure was defined as tumor progression either in or adjacent to the implanted volume. The median local control was 28 months (95% CI 16.2–39.8 months). The percentage of patients who showed 6-month, 1-year, 2-year, and 3-year local control was 97.2%, 77.8%, 52.8%, and 33.3%, respectively (Fig. 1).

**Fig. 2:**
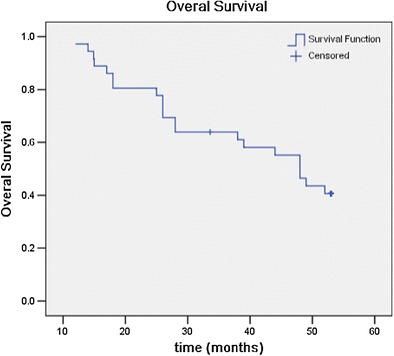
Overall survival for all patients after iodine-125 seed implantation therapy

Twenty-one patients died of distant metastasis. Fifteen patients survived during this follow-up period. Median survival time for all patients was 48 months (95% CI 40.9–55.1 months); 1-year, 2-year, 3-year, and 4-year survival rates were 97.2%, 80.6%, 63.9%, and 46.5%, respectively (Fig. 2).

### Complications and Adverse Reactions

During the follow-up period, no serious complications due to ^125^I seeds were detected. Two patients suffered from localized skin erythema 8 days post implantation, but it disappeared after topical treatment. No ^125^I seeds migrated to other distant tissues or organs. Twenty-eight patients under combination chemotherapy showed varying degrees of leucopenia, liver or renal function impairment. These complications were reversed through symptomatic treatment.

## Discussion

LRBC represents a therapeutic challenge, especially the cases previously treated with multi-modality therapy. Nevertheless, LRBC alone may not always herald a fatal outcome and should not be a predicament for palliative treatment. On the contrary, LRBC treatment strategies should be tolerable, effective, and with long-lasting local control to improve the quality of life. The treatment strategies depend on the type of prior surgery, with or without adjuvant RT [18]. Other variables that could influence the outcome should also be considered: the size and the type of lesion (superficial diffuse or nodular), presence or absence of coexisting metastatic disease, time interval between the first treatment and retreatment, former chemotherapy or endocrine therapy, and other key factors.

Nowadays, surgery, chemotherapy, and EBRT are commonly applied as palliative treatments in LRBC. Local radical resection alone can only be offered to one-third of the patients with recurrence [19, 20], and they achieved a 5-year disease-free survival rate of 45.5% [21]. Although local radical surgery is usually recommended, this may not be feasible in sizeable tumors [22]. Chemotherapy for LRBC has also been explored, but it was not found to be very effective [21]. Repeated EBRT has also limited efficacy in these patients. Reirradiation (ReRT) with full EBRT doses could increase the occurrence of both acute and late morbidities, because most patients with LRBC have received prior RT, thus ReRT could represent a risk of exceeding radiation tolerance limits. For tumors >5 cm, it is difficult to deliver a large enough dose with EBRT, because RT should avoid damaging the adjacent normal tissues such as the lung, spinal cord, and heart [23]. A study reported that for tumors >5 cm, the effective EBRT dose is always greater than 100 Gy [24].

The ^125^I seed implantation method has received more and more attention in view of slight injury to normal tissues. This therapy was initially used for the treatment of prostate cancer and has also been investigated in the treatment of other solid tumors in recent decades [7–10]. There are studies documenting its efficacy in terms of local tumor control [25–27].

In the present retrospective study, we demonstrated that patients with LRBC could benefit from ^125^I seed implantation, given that the method yielded acceptable median survival and progression-free survival times, without any serious complications.

Our results indicate that ^125^I seed implantation exerts the following potential advantages: (1) it has a relatively long half-life, thus extending the radiation effects on the tumor cells; (2) ^125^I seed implantation is not affected by respiratory movements which decrease the therapeutic volume in EBRT; (3) for tumors >5 cm, it delivers a large enough dose of localized radiation due to particle density, leading to tumor shrinkage without serious damage to the adjacent normal tissues; (4) ^125^I seeds have a low risk of side effects [28] and do not cause serious complications such as lymphedema, radiation pneumonitis, and pericarditis; (5) under local anesthesia, the ^125^I seed implantation technique can be performed easily, and the treatment time is shorter; (6) ^125^I seed radiation dose decreases with increasing distance, therefore reducing the damage to the adjacent normal tissues [29–31]. Based on our current results, we conclude that ^125^I seed implantation is clinically effective for locoregionally recurrent and unresectable breast cancers, reducing pain and remarkably improving the patients’ quality of life.

However, our study also has some limitations, due to the retrospective nature of this analysis. First, some patients had to be excluded because their treatment post recurrence course or outcome could not be analyzed; second, we did not have a control group treated with other therapies and with similarly localized tumor recurrence. Finally, our study was based on a small number of patients and a short follow-up time. Therefore, large and well-designed prospective trials are still needed to refine the patient selection criteria, the optimal ^125^I seed implantation dose, re-implantation interval time, and ideal treatment parameters.

## Conclusions

Our results demonstrate that ^125^I seed implantation could be considered as a feasible and promising minimally invasive therapy for locoregionally recurrent and unresectable breast carcinoma, since it appears to be safe, inducing small damage, few adverse effects, slight pain, and can be performed in a short amount of time.

## Abbreviations

CI: Confidence interval
EBRT: External beam radiation therapy
GTV: Gross tumor volume
LRBC: Locoregionally recurrent breast cancer
MPD: Minimal peripheral dose
NRS: Numerical Rating Scale
PTV: Planning treatment volume
ReRT: Reirradiation
RT: Radiation therapy
TPS: Treatment planning system
